# First person – Latifa Bakiri

**DOI:** 10.1242/dmm.052263

**Published:** 2025-01-31

## Abstract

First Person is a series of interviews with the first authors of a selection of papers published in Disease Models & Mechanisms, helping researchers promote themselves alongside their papers. Latifa Bakiri is first author on ‘ A new effLuc/Kate dual reporter allele for tumour imaging in mice’, published in DMM. Latifa is a senior postdoc in the lab of Erwin F. Wagner at Medical University of Vienna, Department of Laboratory Medicine, investigating the functions of the proteins forming the AP-1 transcription factor in physiology and disease by using state-of-the-art genetically engineered mouse models.


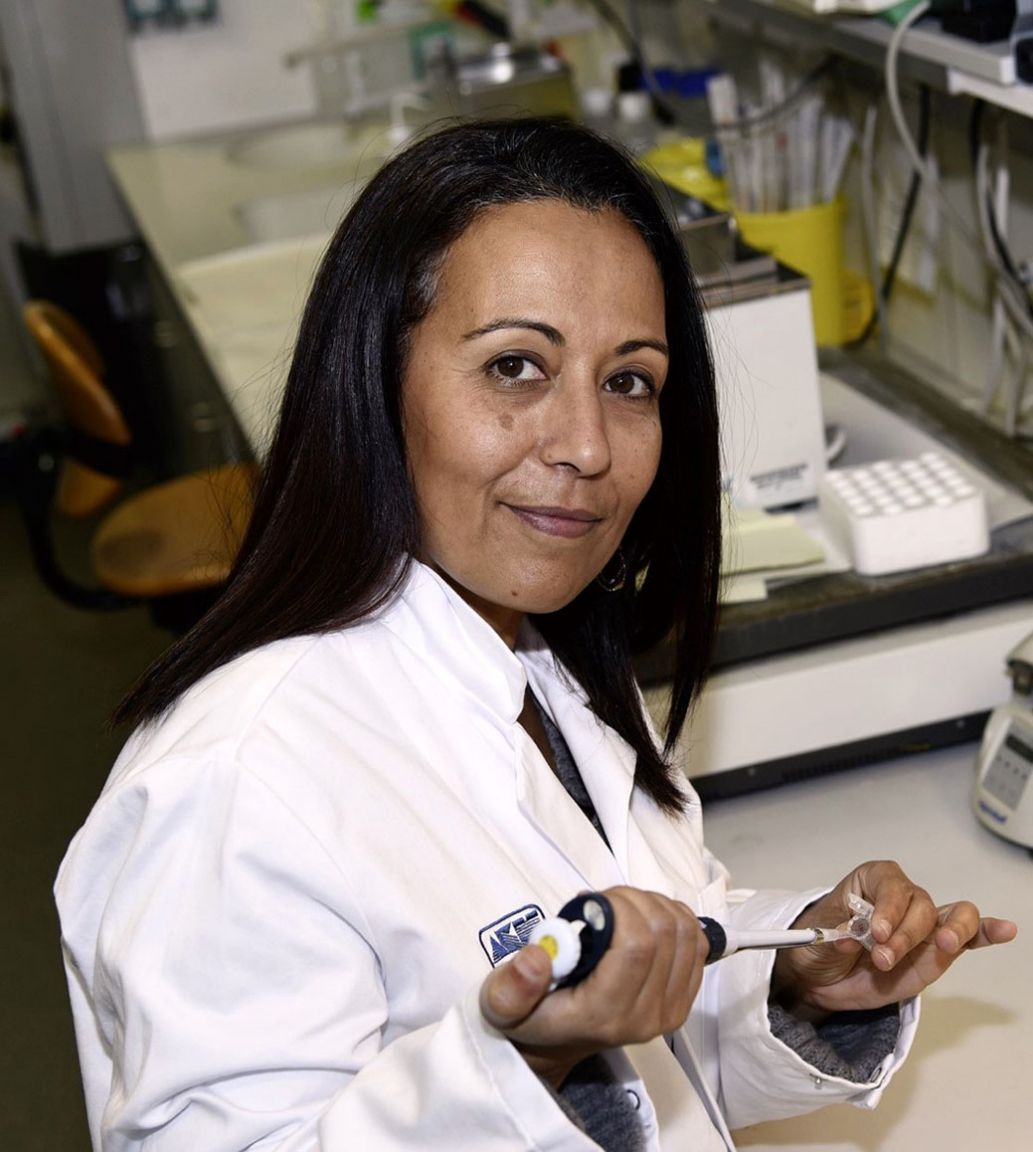


**Latifa Bakiri**

**Who or what inspired you to become a scientist?**

All the fantastic high school science teachers.

**What is the main question or challenge in disease biology you are addressing in this paper? How did you go about investigating your question or challenge?**

We wanted to generate a robust dual (luminescent and fluorescent) reporter allele for non-invasive, longitudinal tumor cell detection and monitoring, which could be shared with the scientific community. We felt that such a tool was needed and would be useful to advance basic and preclinical research.

**How would you explain the main findings of your paper to non-scientific family and friends?**

We made a two-color tool that can mark specific cells of the mouse body, for example, cancer cells. We can then find these cells inside the live mouse because they emit light the same way one would see a person holding a lamp within a crowd. We can also see these marked cells under the microscope inside the organs, even when they are very few because these marked cells are also fluorescent. Finally, this method also allows us to purify the marked cells to study them in detail. This will be very useful for researchers working on tumors and metastasis, and use the mouse as a model system to mimic the body of a patient.

**What are the potential implications of these results for disease biology and the possible impact on patients?**

The new allele will help to better understand the spatiotemporal events driving cancer progression as well as to conduct preclinical studies to evaluate therapies in mouse models. Advancing basic and preclinical research is beneficial to the patients in the long term.

We wanted a journal known to publish resources, such as mouse models and alleles, that are used to advance basic and preclinical research. Therefore, DMM was ideal.

**Figure:**
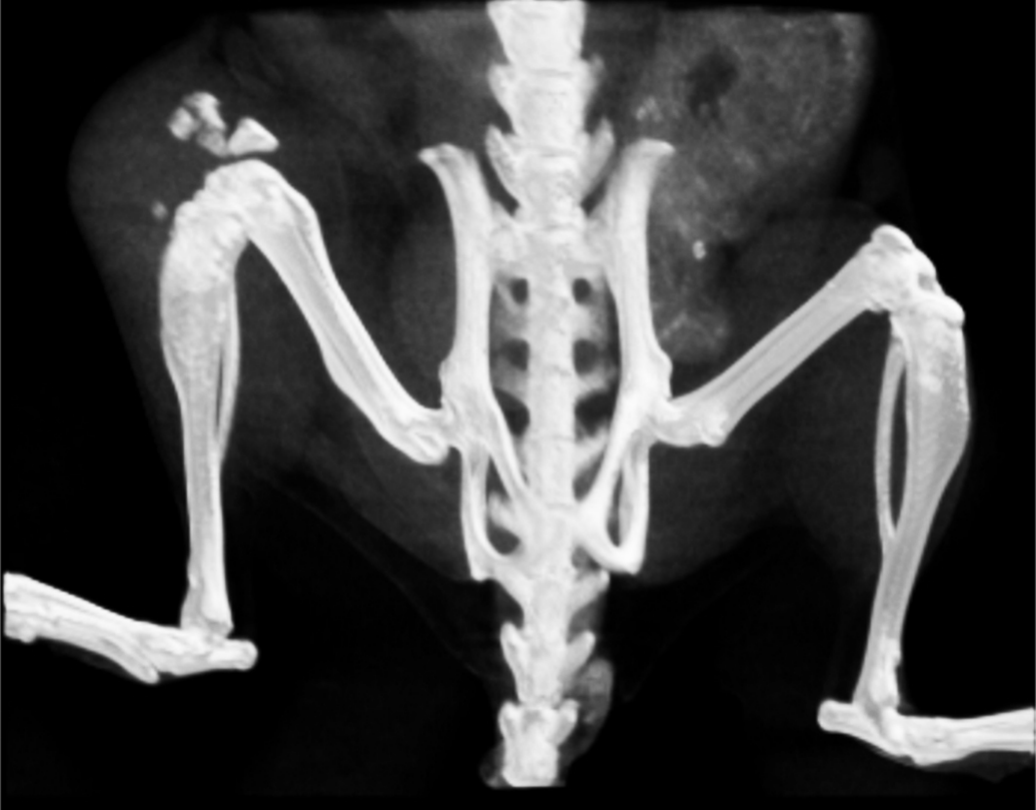
**3D reconstitution of the hind legs of an adult mouse subjected to microCT.** Small ectopic calcifications and bone destruction due to a soft tissue tumor can be observed around the right knee.

**Why did you choose DMM for your paper?**

We were looking for an Open Access research journal with a rigorous but also reasonably fast peer-review process. We wanted a journal known to publish resources, such as mouse models and alleles, that are used to advance basic and preclinical research. Therefore, DMM was ideal.

We also like The Company of Biologists as a not-for-profit publishing organization run by researchers for researchers. In the past, The Company of Biologists has supported a scientific meeting organized by us and is known to support early-stage researchers.

It would be nice if more funding would be available for small research projects with little preliminary data and if journals would be more professional in handling manuscripts.

**Given your current role, what challenges do you face and what changes could improve the professional lives of other scientists in this role?**

The challenges are mostly related to the amount of time that I have to spend on administrative tasks, on grant writing and submission and, to a lesser extent, on manuscript writing and reviewing. It would be nice if more funding would be available for small research projects with little preliminary data and if journals would be more professional in handling manuscripts. On one hand, we often have to wait weeks for an editor to just decide if she/he will send the manuscript for review or not while, on the other hand, we sometimes receive requests to review manuscripts that do not meet minimal editorial or even formatting standards. Publishing is paramount to success in grant applications and career advancement.

**What's next for you?**

Writing my next grant proposal − and my next paper!

**Tell us something interesting about yourself that wouldn't be on your CV**

If not science, I would probably have worked in something related to literature. I love books!

**If you would like to add a question of your own, enter it here**

How do you see the future of scientific publishing? My answer would be: all for free (for author and reader) and always open access but − of course − with high-quality peer review.
